# Validity and Reliability of the Polish Adaptation of the CHAMPS Physical Activity Questionnaire

**DOI:** 10.1155/2019/6187616

**Published:** 2019-03-28

**Authors:** Magdalena Król-Zielińska, Monika Ciekot-Sołtysiak, Robert Szeklicki, Jacek Zieliński, Wiesław Osiński, Adam Kantanista

**Affiliations:** ^1^Department of Physical Education and Lifelong Sports, Faculty of Physical Education, Sport and Rehabilitation, Poznan University of Physical Education, Królowej Jadwigi 27/39, 61-871 Poznań, Poland; ^2^Department of Athletics Strength and Conditioning, Faculty of Physical Education, Sport and Rehabilitation, Poznan University of Physical Education, Królowej Jadwigi 27/39, 61-871 Poznań, Poland; ^3^Department of Physical Activity Science and Health Promotion, Faculty of Physical Education, Sport and Rehabilitation, Poznan University of Physical Education, Królowej Jadwigi 27/39, 61-871 Poznań, Poland

## Abstract

The aim of the study was to investigate the reliability and construct validity of the Polish adaptation of the Community Health Activities Model Program for Seniors (CHAMPS) physical activity questionnaire among the elderly. The sample included 104 volunteers, 75 women (age = 71.0 ± 5.0 years) and 29 men (age = 75.1 ± 6.6 years). To assess the reliability of the Polish version of the CHAMPS physical activity questionnaire, measurements were conducted by one-week test-retest. The construct validity of the CHAMPS physical activity questionnaire was evaluated using accelerometers. Criterion validation was verified by self-reported measurements (health self-assessment, life satisfaction, and wellbeing) and body composition analysis. Intraclass correlation coefficients of the one-week test-retest ranged from 0.79 to 0.85. Significant Pearson's correlations were found between caloric expenditure measured by accelerometer and CHAMPS caloric expenditure in all listed physical activities (*r* = 0.33) and caloric expenditure in at least moderate intensity physical activities (*r* = 0.37) of the CHAMPS physical activity questionnaire. Moderate and greater intensity physical activities of CHAMPS measure were significantly related to total bone mass, health self-assessment, life satisfaction as a whole, and personal wellbeing (*r* ranged from 0.26 to 0.34). The findings of the study allow us to conclude that the Polish version of the CHAMPS physical activity questionnaire has acceptable reliability and validity to assess physical activity of older adults.

## 1. Introduction

It has been found in many studies that physical activity is associated with better health in the elderly (e.g., [1–3]) and sedentary behaviors are associated with all-cause mortality, metabolic syndrome, waist circumference, and obesity [4, 5]. Because of aging of the world's population, research on physical activity in the elderly and the effectiveness of physical activity interventions is important. Measurement of physical activity is necessary to indicate the desired amount of physical activity and to understand the mechanisms of the relationship between physical activity and health [6].

Questionnaires are the most commonly used tool in epidemiological studies and large-scale trials. Self-report data has some limitations, among others resulting from recall bias (defined as differences in the accuracy of recall across comparable groups [7]) and the “floor” effect (which arises when more than 15% of people who completed the questionnaire achieved the lowest possible score [8]) but assessing physical activity with questionnaires is easily accessible, noninvasive, and not expensive [9, 10]. The questionnaire on physical activity for the elderly should take into account the specific physical activity at this age.

Among different questionnaires, the Community Health Activities Model Program for Seniors (CHAMPS) physical activity questionnaire allows evaluation of the types and intensity levels of physical activity that are meaningful and appropriate for older adults [11]. The CHAMPS physical activity questionnaire is one of the most valid and reliable questionnaires for assessing physical activity of elderly people [10, 12] and could make the measure valuable for use in epidemiologic and intervention studies [13].

The reliability and validity of the CHAMPS measure were examined in different populations and the findings of the studies suggested that the CHAMPS physical activity questionnaire had acceptable measurement properties and is an appropriate tool for physical activity assessment in older adults [13–15]. Test-retest reliability coefficients for the CHAMPS all activity measures were 0.62 (Pearson's correlation and intraclass correlation coefficients (ICC)) [10]. In the study by Hekler et al. [13], the CHAMPS different activities (sedentary, low-light, high-light, total activity, and moderate to vigorous physical activity (MVPA) variables) had an acceptable test-retest reliability (ICCs ranged from 0.56 to 0.70). The validity of the CHAMPS physical activity questionnaire was usually assessed using accelerometers [13, 16], fitness tests [10, 14], pedometers [15], activity monitors, and health quality of life [10], but not with body composition analysis. It has been shown that lower body mass index (BMI) and fat mass were associated with higher physical activity [17]. A study assessing the validity of CHAMPS measure using accelerometers found that the CHAMPS high-light (*ρ* = 0.27), total activity (*ρ* = 0.34), and MVPA (*ρ* = 0.37) duration scales were moderately associated with accelerometry time (minutes) of corresponding intensity [13]. In another study [10], Pearson's correlations of CHAMPS measure with Mini-Logger counts (on the waist) were 0.59 in male and 0.31 in female older adults.

Identification and choice of measurement tools enable a valid and reliable assessment of physical activity. It is essential in the process of collecting evidence-based data. Most physical activity questionnaires have been developed for youth and adults and fewer for the elderly population [18]. To date, there is a lack of reliable and validated physical activity questionnaires adapted to the Polish population above 69 years old and research on physical activity requires methodological improvement [19]. Based on studies of the Polish population, it is not possible to answer questions reliably about physical activity levels of Poles and the percentage of those who meet the World Health Organization recommendations on physical activity [18]. For the elderly of the Polish population, only the International Physical Activity Questionnaire was adapted, but it is designed for people aged 15–69 years [20].

The aims of the present study were (i) to assess the construct validity of the CHAMPS physical activity questionnaire using accelerometers, (ii) to assess the criterion validity of the CHAMPS with body composition analysis and following self-reported measures: health self-assessment, life satisfaction, and personal wellbeing, and (iii) to assess the test-retest reliability.

It was hypothesized that the CHAMPS physical activity questionnaire is an accurate tool of assessment of physical activity of the Polish elderly population and values of validity and reliability indicators would be similar to those obtained in previous studies.

## 2. Material and Methods

### 2.1. Participants and Procedure

In the reliability study of the Polish version of the CHAMPS physical activity questionnaire (test-retest within one week), 104 older adults (75 women and 29 men) were included. In the validity measurement, 79 elderly adults (59 women and 20 men) participated. The participants were recruited as volunteers through announcements and invited to an information meeting. A notice was posted in the local newspaper, websites of the Poznan University of Physical Education and municipal organization for seniors (The Center for Senior Citizens Initiatives), and leaflets were distributed in places where the elderly can most often be found (e.g., pharmacy). People aged at least 65 years and able to move independently were eligible to participate in the study. Participants were presented with detailed research objectives and methods of measurement, and then it was explained how to complete the questionnaires. They could ask for help while completing the surveys at every stage of the research. Participants were also trained in the issue of setting up accelerometers. The study was approved by the ethics committee at the Poznan University of Medical Sciences (971/12), and all participants gave their informed and written consent.

### 2.2. The CHAMPS Physical Activity Questionnaire

The CHAMPS physical activity questionnaire assesses weekly frequency and duration of various physical activities. The questionnaire includes light as well as more vigorous activities. The CHAMPS measure also takes into consideration sedentary behaviors (e.g., sitting). Respondents must recall the type and frequency of physical activities undertaken during one typical week from the past 4 weeks. An example of a questionnaire item is the following: “In a typical week during the past 4 weeks, did you walk fast or briskly for exercise?” The participant indicates how many times this activity was performed in a week and then considers the total hours per week spent on this activity and chooses one of the six response options, ranging from “less than one hour” to “9 or more hours.” Caloric expenditure and frequency are generated for all activities (any MET value) and for at least moderate intensity activities (MET value ≥3.0). The MET value was assigned to each form of physical activity included in the questionnaire based on data presented in the work of Ainsworth et al. [21]. Energy expenditure was estimated by multiplying the approximate time devoted to each form of physical activity by the appropriate MET value and summing up the results obtained for all types of activities [11].

### 2.3. Polish Adaptation Procedure of the CHAMPS Physical Activity Questionnaire

The theoretical structure of the CHAMPS physical activity questionnaire was analyzed by two independent scientists in the field of physical activity methodology. They analyzed the theoretical background of the CHAMPS physical activity questionnaire and differences in Polish culture and language used in the original version of the survey. Subsequently, the adaptation procedure consisted of translation by two bilingual persons, review and comparison of translations, discussion, and a unified draft version. Next, the questionnaire was back-translated by two other bilingual persons. Then, the revision and comparison procedure was repeated and a unified final Polish version was formed (see Supplementary materials (available here)). Finally, quantitative research of the adapted version of the questionnaire was conducted, including assessment of time stability, and construct and criterion validity assessment.

To assess the measurement reliability of the adapted version of the questionnaire in terms of the stability aspect, recurrent measurements were performed (repeated measurement with the use of the same test-test-retest within 1-week-time stability). During completion of the questionnaire, a research assistant was available to help participants.

To verify construct validity, results of physical activity obtained with the use of the adapted CHAMPS physical activity questionnaire were compared with physical activity assessment performed with the use of the ActiGraph model wGT3X+ (ActiGraph, LLC, Pensacola, FL, USA). Participants wore an ActiGraph accelerometer on the right anterior superior iliac spine (on the belt around the waist) for seven consecutive days (all day, except sleeping time, bathing, showering, or swimming) [22, 23]. The accelerometers were programmed to record data in 10-s time epochs. After 7 days, the data was downloaded to the computer and analyzed using the ActiLife6 Analysis Software Suite (ActiGraph, LLC, Pensacola, FL, USA). The weekly energy expenditure, step counts, minutes per week spent in sedentary, light, moderate, vigorous, and very vigorous activity were calculated using Freedson's equation [24]. The CHAMPS physical activity questionnaire was filled in on the day following the 7-day measurement of physical activity with the accelerometer.

Relationships between physical activity levels as measured by the Polish version of the CHAMPS physical activity questionnaire, and life satisfaction and personal wellbeing (PWI-A) [25], health self-assessment (one question, answers on a 5-point scale), and body composition analysis by the X-ray absorptiometry method (DXA) utilizing Lunar Prodigy Pro (GE Healthcare, Madison, WI, USA) were examined. All DXA scans were performed using enCORE 16 SP1 software. The subjects' body mass and height were measured using a digital stadiometer (SECA 285, SECA, Hamburg, Germany).

### 2.4. Statistical Analyses

Basic statistical methods were used to describe continuous variables (mean, standard deviation, and range) and for categorical variables (percentage distribution). To assess the reliability and validity of indicators of the Polish adaptation of the CHAMPS physical activity questionnaire, ICCs for reliability and Pearson's correlations for validity were calculated. The significance level for the Pearson correlation coefficient was set at* p* < 0.05. Cohen's classification [26] was used to interpret the strength of association, according to which the value of the correlation coefficient was defined as small (0.1), medium (0.3), or large (0.5). ICC analyses were performed using SPSS v.20.0 (IBM Corp., Armonk, NY, USA) and all the other analyses were conducted using STATISTICA 13 software (StatSoft, Inc., USA).

## 3. Results

Characteristics of the basic demographic variables of the study participants are presented in Table 1. The sample included 104 persons aged 65–89 years, 75 women (age = 71.0 ± 5.0 years, body height = 1.59 ± 0.06 m, body weight = 67.8 ± 11.9 kg, and BMI = 26.7 ± 4.0 kg·m^−2^) and 29 men (age = 75.1 ± 6.6 years, body height = 1.70 ± 0.06 m, body weight = 80.3 ± 9.6 kg, and BMI = 27.0 ± 3.9 kg·m^−2^). The group was mostly married (52.9%), fairly well educated (45.6%), and lived in a big city (65.4%).

Table 2 presents descriptive statistics and reliability of the CHAMPS measures at test (baseline) and retest. ICC coefficients of test-retest ranged from 0.79 to 0.85 for all CHAMPS physical activity questionnaire outcomes. The highest repeatability rates were obtained in frequency per week in at least moderate physical activities (ICC = 0.85) and frequency per week in all listed physical activities (ICC = 0.84).

Table 3 shows the results of a validity study of the Polish version of the CHAMPS physical activity questionnaire. The energy expenditure during the weekly measurement of physical activity using accelerometers was compared with the physical activity assessed by CHAMPS measure in a typical week in the past 4 weeks. Correlation coefficients were* r* = 0.33 between caloric expenditure measured by accelerometer and by CHAMPS all listed physical activities and* r* = 0.37 between the accelerometer and CHAMPS in at least moderate intensity physical activities. The MVPA indicator calculated on the basis of an accelerometer correlated at the level of* r* = 0.31 with CHAMPS frequency per week in at least moderate intensity physical activities and* r* = 0.24 for CHAMPS caloric expenditure per week in at least moderate intensity physical activities. Time of moderate activity measured by the accelerometer for 7 days was related to CHAMPS frequency per week in at least moderate intensity physical activities (*r* = 0.31) and related to CHAMPS caloric expenditure per week in at least moderate intensity physical activities (*r* = 0.23).

To test the criterion validity of the CHAMPS measure, the results of physical activity and body composition were compared. Total bone mass was significantly related (*r* = 0.26) to moderate and greater intensity physical activities (frequency and caloric expenditure) measured by the CHAMPS physical activity questionnaire. Similarly, health self-assessment, life satisfaction as a whole, and personal wellbeing were also positively correlated with the CHAMPS physical activity questionnaire in at least moderate intensity physical activities (*r* ranged from 0.27 to 0.34).

## 4. Discussion

This study provided information concerning the reliability and validity of the CHAMPS physical activity questionnaire in a sample of elderly people from Poland. To date, the psychometric properties of this questionnaire have not been assessed in the Polish population. Initial evidence suggests that the CHAMPS physical activity questionnaire is an acceptable tool to assess physical activity in the elderly Polish population but there were also some inconsistencies.

A large correlation (one-week test-retest) was observed in frequency and caloric expenditure per week in physical activities (ICCs ranged from 0.79 to 0.85) and may be accepted in the context of using the CHAMPS measure in the elderly population in Poland. Similar reliability coefficients were obtained in the Harada et al. study [10] but in a two-week test-retest, i.e., 0.76 for moderate or greater intensity activities measure and 0.62 for all activities. Cyarto et al. [14] in the study on reliability of the CHAMPS physical activity questionnaire in elderly Australians used a one-week timeframe for test-retest and observed higher ICC coefficients for moderate intensity measures than for vigorous-intensity measures of physical activity. They indicated that one-week test-retest is a better approach in contemporary reliability studies. In addition, Giles and Marshall [15] assessed the reliability and validity of the CHAMPS measure in Australian older adults. They modified the questionnaire and asked participants to recall the activities they had undertaken in the past seven days. Reliability coefficients were good to excellent for all physical activity constructs. In the present study, we found higher coefficients for the frequency measures than for the caloric expenditure measures, which may suggest a better recall of the activities than an evaluation of the type of activities. This result is compatible with the observation in the study by Stewart et al. [11]. The differences in some reliability coefficients may be related to different methodologies of questionnaire administration. In our study, participants were helped by research assistants during all measures.

The construct validity of the CHAMPS physical activity questionnaire was assessed using accelerometer data. Correlations between different physical activity indicators assessed by CHAMPS measure and data from accelerometers expressed in kcal per week, as moderate activity (time), as vigorous activity (time), as step counts, and MVPA were small to medium (*r* ranged from 0.22 to 0.37) and were lower than in Harada et al.'s [10] and Giles and Marshall's [15] studies. However, they used a Mini-Logger Series 2000 monitor, which measured activity by counting the number of mercury switch closures or pedometers, which measured step counts. These two tools are not as accurate in assessing physical activity intensity as accelerometers. In validation studies by Hekler et al. [13] and Cancela et al. [16], where accelerometers were used, correlation coefficients comparable to ours were reported.

Because of evidence that physical activity is often correlated with other health indicators [2], we also examined correlation coefficients between the physical activity measures and body composition, and following self-reported measures: health self-assessment, life satisfaction as a whole, and personal wellbeing.

Total lean mass and total fat mass did not correlate with physical activity measured by the CHAMPS physical activity questionnaire. Our expectation was not confirmed. To our knowledge, in other validation and reliability studies body composition analysis was not included. However, BMI in some studies was assessed [10, 11] and a correlation between the BMI and physical activity evaluated by the CHAMPS measure was not observed. It should be noted that participants in our study were volunteers, which could be more physically active than the general population. It might be significant in determining the level of the relationship between physical activity and body composition. Nevertheless, it seems that empirical evidence concerning the relationship between BMI and physical activity is still inconclusive. In our study, total bone mass was positively associated with frequency and caloric expenditure per week in at least moderate intensity physical activities. It is consistent with results of other studies, which indicated that physical activity may improve total bone mass, bone mineral content, and bone mineral density and reduce the risk of bone fractures related to falls [27, 28].

We observed small to medium correlations (*r* ranged from 0.23 to 0.34) of frequency and caloric expenditure per week in at least moderate intensity physical activities with self-reported measures. There were no such associations with frequency and caloric expenditure per week in all listed physical activities. Our observation is partly consistent with the results of other authors. Stewart et al. [11] found a small positive correlation between the self-reported psychological wellbeing and only frequency per week in at least moderate intensity physical activities. In the study by Harada et al. [10], four validation measures concerning health status were included. The correlations between all physical activity measures using the CHAMPS physical activity questionnaire and physical functioning and general health measures were higher (*r* ranged from 0.26 to 0.42) than correlations with the mental health and pain scores (*r* ranged from 0.17 to 0.28). In contrast to our results, Cyarto et al. [14] found no correlation between the CHAMPS physical activity questionnaire and mental components of health.

The following limitations should be considered when interpreting the findings of this study. First, the older adults voluntarily took part in the study. Usually, volunteers are more physically and socially active, so they do not fully reflect the entire population of older people. Second, the sample size was relatively small and mostly women. This could limit the generalizability of the findings. Third, completing the questionnaire the first time could increase awareness of physical activity. It could influence the recall of physical activity during subsequent completing of questionnaires after one week and in consequence potentially influence reliability.

The current study also has several strengths. The construct validity of the CHAMPS physical activity questionnaire was assessed using accelerometers. Despite some limitations of accelerometers, it is an objective method of physical activity evaluation. To assess the criterion validity of the CHAMPS physical activity questionnaire, the DXA method was used to assess body composition. This method fulfills the highest criteria of accuracy. In addition, during completing of the CHAMPS measure a research assistant was available to help participants in the case of lack of understanding the instructions or questions.

## 5. Conclusions

The findings of this study are in major part consistent with the results observed previously and allow us to conclude that the Polish version of the CHAMPS physical activity questionnaire has acceptable reliability and validity to assess physical activity of older adults.

## Figures and Tables

**Table 1:** Demographic characteristics of the participants.

	Total	Women	Men
	(N = 104)	(N = 75)	(N = 29)
Age (yr)*∗*	72.2 (5.7)	71.0 (5.0)	75.1 (6.6)
Body height (m)*∗*	1.62 (0.08)	1.59 (0.06)	1.70 (0.06)
Body weight (kg)*∗*	71.3 (12.6)	67.8 (11.9)	80.3 (9.6)
Body Mass Index (kg·m^–2^)*∗*	27.0 (3.9)	26.7 (4.0)	27.8 (3.6)
Marital status†			
Married	55 (52.9)	31 (41.3)	24 (82.8)
Widow/Widower	32 (30.8)	29 (38.7)	3 (10.3)
Divorced	10 (9.6)	8 (10.7)	2 (6.9)
Unmarried	7 (6.7)	7 (9.3)	0
Education†			
Higher	47 (45.6)	31 (41.9)	16 (55.2)
Secondary	35 (34.0)	30 (40.5)	5 (17.2)
Vocational	13 (12.6)	6 (8.1)	7 (24.1)
Primary	8 (7.8)	7 (9.5)	1 (3.5)
Place of residence†			
Less than 20 000 inhabitants	22 (21.1)	16 (21.3)	6 (20.7)
20 000–500 000 inhabitants	14 (13.5)	10 (13.3)	4 (13.8)
More than 500 000 inhabitants	68 (65.4)	49 (65.4)	19 (65.5)

*∗*Mean (standard deviation)

† N (%).

**Table 2:** Test and retest results of CHAMPS physical activity questionnaire, descriptive statistics and correlations.

CHAMPS physical activity questionnaire	Test		Retest		ICC
	Mean (SD)	Range	Mean (SD)	Range	
Frequency per week in all listed physical activities	15.3 (7.3)	2–39	15.1 (7.2)	2–38	0.84
Caloric expenditure per week in all listed physical activities (kcal·week^−1^)	3693 (2357)	175–10683	3619 (2263)	175–13514	0.80
Frequency per week in at least moderate intensity physical activities	6.0 (4.5)	0–22	5.7 (4.8)	0–20	0.85
Caloric expenditure per week in at least moderate intensity physical activities (kcal·week^−1^)	1731 (1499)	0–6837	1724 (1717)	0–8247	0.79

**Table 3:** Correlations between physical activity measured by the CHAMPS physical activity questionnaire and accelerometer, body composition, aerobic endurance, and self-reported variables.

	CHAMPS physical activity questionnaire			
	Frequency per week in all listed physical activities	Caloric expenditure per week in all listed physical activities	Frequency per week in at least moderate intensity physical activities	Caloric expenditure per week in at least moderate intensity physical activities
Accelerometer				
Kcal per week	0.20	0.33*∗*	0.34*∗*	0.37*∗*
Moderate activity (time)	0.27*∗*	0.19	0.31*∗*	0.23*∗*
Vigorous activity (time)	0.31*∗*	0.22*∗*	0.29*∗*	0.22
Step counts	0.26*∗*	0.18	0.32*∗*	0.24*∗*
MVPA	0.27*∗*	0.20	0.31*∗*	0.24*∗*
Body composition				
Total lean mass	–0.03	0.12	0.19	0.19
Total fat mass	–0.03	0.22	–0.05	0.13
Total bone mass	–0.04	0.12	0.26*∗*	0.26*∗*
Health self-assessment	0.19	0.14	0.32*∗*	0.28*∗*
Satisfaction with life as a whole	0.15	0.17	0.27*∗*	0.29*∗*
Personal wellbeing	0.08	0.10	0.30*∗*	0.34*∗*

*∗p* < 0.05.

## Data Availability

The data used to support the findings of this study are available from the corresponding author upon request.

## Supplementary Materials

Supplementary Materials Supplementary materials contain Polish version of the CHAMPS physical activity questionnaire.
