# Supplementary material for: Validity and Reliability of the Polish Adaptation of the CHAMPS Physical Activity Questionnaire
Source: Biomed Res Int. 2019 Mar 28;2019:6187616. doi: 10.1155/2019/6187616 (PMC6458940; doi:10.1155/2019/6187616)
Supplement: Supplementary Materials — contain Polish version of the CHAMPS physical activity questionnaire. [file 6187616.f1.zip › 6187616.f1/Supplementary materials_BMRI_2704614.pdf]

CHAMPS - KWESTIONARIUSZ AKTYWNOŚCI DLA OSÓB STARSZYCH W POLSKIEJ ADAPTACJI

Oryginalna wersja:
CHAMPS: Community Healthy Activities Model Program for Seniors
Institute for Health & Aging, Center for Healthy and Active Aging
University of California, San Francisco
Stanford Center for Research in Disease Prevention, Stanford University

Data: ____________________________

Nazwisko lub ID: _________________

Ten kwestionariusz dotyczy aktywności, które mógł/a Pan/i podejmować w ciągu ostatnich 4 tygodni. Pytania na kolejnych stronach są podobne do poniższego przykładu.

INSTRUKCJA

Jeśli PODEJMOWAŁ/A Pan/i określoną aktywność w ciągu ostatnich 4 tygodni:

Krok nr 1 **Proszę wybrać odpowiedź tak.**

Krok nr 2 **Proszę pomyśleć, <u>ile</u> RAZY <u>w ciągu tygodnia</u> zwykle podejmował/a Pan/i tę aktywność, a następnie wpisać odpowiedź w wyznaczonym miejscu.**

Krok nr 3 **Proszę zaznaczyć, ile GODZIN OGÓŁEM poświęcał/a Pan/i na tę aktywność <u>w ciągu typowego tygodnia</u>.**

Poniżej przedstawiono przykład odpowiedzi, jaką mogła udzielić Pani Kowalska na Pytanie nr 1:
Pani Kowalska zwykle odwiedza swoje znajome, Marię i Olgę, <u>dwa razy w tygodniu</u>. Zwykle spędza u Marii <u>1 godzinę</u> w poniedziałek, a w środę przebywa przez <u>2 godziny</u> u Olgi. Zatem ogółem pani Kowalska poświęca na spotkania ze znajomymi <u>3 godziny</u> tygodniowo.

Czy w ciągu typowego tygodnia podczas ostatnich 4 tygodni…							
1. Odwiedzał/a Pan/i znajomych lub krewnych (z wyłączeniem tych, z którymi Pan/i mieszka)?<br>☒TAK Ile razy w ciągu tygodnia? 2 ➧<br>☐ NIE	Ile OGÓŁEM <u>godzin w tygodniu</u> zwykle poświęcał/a Pan/i na to? ➧	Mniej niż 1 godz.	1-2,5 godz.	(3-4,5 godz.)	5-6,5 godz.	7-8,5 godz.	9 i więcej godz.

Jeśli NIE PODEJMOWAŁ/A Pan/i danej aktywności:

- **Proszę zaznaczyć odpowiedź NIE i przejść do kolejnego pytania**

Czy w ciągu typowego tygodnia podczas ostatnich 4 tygodni…							
1. Odwiedzał/a Pan/i znajomych lub krewnych (z wyłączeniem tych, z którymi Pan/i mieszka)?<br>☐ TAK Ile razy w ciągu tygodnia?____➧<br>☐ NIE	Ile OGÓŁEM <u>godzin w tygodniu</u> zwykle poświęcał/a Pan/i na to? ➧	Mniej niż 1 godz.	1-2,5 godz.	3-4,5 godz.	5-6,5 godz.	7-8,5 godz.	9 i więcej godz.
2. Była Pan/i w ośrodku dla seniorów?<br>☐ TAK Ile razy w ciągu tygodnia?____➧<br>☐ NIE	Ile OGÓŁEM <u>godzin w tygodniu</u> zwykle poświęcał/a Pan/i na to? ➧	Mniej niż 1 godz.	1-2,5 godz.	3-4,5 godz.	5-6,5 godz.	7-8,5 godz.	9 i więcej godz.
3. Wykonywał/a Pan/i prace społeczne (wolontariat)?<br>☐ TAK Ile razy w ciągu tygodnia?____➧<br>☐ NIE	Ile OGÓŁEM <u>godzin w tygodniu</u> zwykle poświęcał/a Pan/i na to? ➧	Mniej niż 1 godz.	1-2,5 godz.	3-4,5 godz.	5-6,5 godz.	7-8,5 godz.	9 i więcej godz.

4. Był/a Pan/i w kościele lub uczestniczył/a Pan/i w pracach kościelnych?<br>☐ TAK Ile razy w ciągu tygodnia?____➧<br>☐ NIE	Ile OGÓŁEM <u>godzin w tygodniu</u> zwykle poświęcał/a Pan/i na to? ➧	Mniej niż 1 godz.	1-2,5 godz.	3-4,5 godz.	5-6,5 godz.	7-8,5 godz.	9 i więcej godz.
5. Był/a Pan/i na spotkaniu jakiegoś klubu lub grupy?<br>☐ TAK Ile razy w ciągu tygodnia?____➧<br>☐ NIE	Ile OGÓŁEM <u>godzin w tygodniu</u> zwykle poświęcał/a Pan/i na to? ➧	Mniej niż 1 godz.	1-2,5 godz.	3-4,5 godz.	5-6,5 godz.	7-8,5 godz.	9 i więcej godz.
6. Używał/a Pan/i komputera?<br>☐ TAK Ile razy w ciągu tygodnia?____➧<br>☐ NIE	Ile <u>godzin w tygodniu</u> OGÓŁEM zwykle Pan/i poświęcał/a na to?	Mniej niż 1 godz.	1-2,5 godz.	3-4,5 godz.	5-6,5 godz.	7-8,5 godz.	9 i więcej godz.
7. Tańczył/a Pan/i (tj. taniec w parach, w grupie, taniec ludowy, towarzyski) (<u>z wyłączeniem</u> aerobiku tanecznego)?<br>☐ TAK Ile razy w ciągu tygodnia?____➧<br>☐ NIE	Ile OGÓŁEM <u>godzin w tygodniu</u> zwykle poświęcał/a Pan/i na to? ➧	Mniej niż 1 godz.	1-2,5 godz.	3-4,5 godz.	5-6,5 godz.	7-8,5 godz.	9 i więcej godz.
8. Zajmował/a się Pan/i stolarką, robótkami ręcznymi, rysowaniem lub inną sztuką czy rzemiosłem?<br>☐ TAK Ile razy w ciągu tygodnia?____➧<br>☐ NIE	Ile OGÓŁEM <u>godzin w tygodniu</u> zwykle poświęcał/a Pan/i na to? ➧	Mniej niż 1 godz.	1-2,5 godz.	3-4,5 godz.	5-6,5 godz.	7-8,5 godz.	9 i więcej godz.
9. Grał/a Pan/i w golfa, nosząc lub ciągnąc swój sprzęt golfowy (proszę policzyć wyłącznie czas przeznaczony na <u>chodzenie</u>)?<br>☐ TAK Ile razy w ciągu tygodnia?____➧<br>☐ NIE	Ile OGÓŁEM <u>godzin w tygodniu</u> zwykle poświęcał/a Pan/i na to? ➧	Mniej niż 1 godz.	1-2,5 godz.	3-4,5 godz.	5-6,5 godz.	7-8,5 godz.	9 i więcej godz.
10. Grał/a Pan/i w golfa, jeżdżąc wózkiem golfowym (proszę policzyć wyłącznie czas przeznaczony na <u>chodzenie</u>)?<br>☐ TAK Ile razy w ciągu tygodnia?____➧<br>☐ NIE	Ile OGÓŁEM <u>godzin w tygodniu</u> zwykle poświęcał/a Pan/i na to? ➧	Mniej niż 1 godz.	1-2,5 godz.	3-4,5 godz.	5-6,5 godz.	7-8,5 godz.	9 i więcej godz.
11. Był/a Pan/i na koncercie, filmie, wykładzie lub wydarzeniu sportowym?<br>☐ TAK Ile razy w ciągu tygodnia?____➧<br>☐ NIE	Ile OGÓŁEM <u>godzin w tygodniu</u> zwykle poświęcał/a Pan/i na to? ➧	Mniej niż 1 godz.	1-2,5 godz.	3-4,5 godz.	5-6,5 godz.	7-8,5 godz.	9 i więcej godz.
12. Grał/a Pan/i w karty, bingo lub gry planszowe z innymi osobami?<br>☐ TAK Ile razy w ciągu tygodnia?____➧<br>☐ NIE	Ile OGÓŁEM <u>godzin w tygodniu</u> zwykle poświęcał/a Pan/i na to? ➧	Mniej niż 1 godz.	1-2,5 godz.	3-4,5 godz.	5-6,5 godz.	7-8,5 godz.	9 i więcej godz.
13. Grał/a Pan/i w bilard?<br>☐ TAK Ile razy w ciągu tygodnia?____➧<br>☐ NIE	Ile OGÓŁEM <u>godzin w tygodniu</u> zwykle poświęcał/a Pan/i na to? ➧	Mniej niż 1 godz.	1-2,5 godz.	3-4,5 godz.	5-6,5 godz.	7-8,5 godz.	9 i więcej godz.

14. Grał/a Pan/i w tenisa w grę pojedynczą (z wyłączeniem gry podwójnej, deblowej)?<br>☐ TAK Ile razy w ciągu tygodnia?____➡<br>☐ NIE	Ile OGÓŁEM godzin w tygodniu zwykle poświęcał/a Pan/i na to? ➡	Mniej niż 1 godz.	1-2,5 godz.	3-4,5 godz.	5-6,5 godz.	7-8,5 godz.	9 i więcej godz.
15. Grał/a Pan/i w tenisa w grę podwójną (z wyłączeniem gry pojedynczej, singlowej)?<br>☐ TAK Ile razy w ciągu tygodnia?____➡<br>☐ NIE	Ile OGÓŁEM godzin w tygodniu zwykle poświęcał/a Pan/i na to? ➡	Mniej niż 1 godz.	1-2,5 godz.	3-4,5 godz.	5-6,5 godz.	7-8,5 godz.	9 i więcej godz.
16. Jeździł/a Pan/i na łyżwach, wrotkach, rolkach?<br>☐ TAK Ile razy w ciągu tygodnia?____➡<br>☐ NIE	Ile OGÓŁEM godzin w tygodniu zwykle poświęcał/a Pan/i na to? ➡	Mniej niż 1 godz.	1-2,5 godz.	3-4,5 godz.	5-6,5 godz.	7-8,5 godz.	9 i więcej godz.
17. Grał/a Pan/i na instrumencie muzycznym?<br>☐ TAK Ile razy w ciągu tygodnia?____➡<br>☐ NIE	Ile OGÓŁEM godzin w tygodniu zwykle poświęcał/a Pan/i na to? ➡	Mniej niż 1 godz.	1-2,5 godz.	3-4,5 godz.	5-6,5 godz.	7-8,5 godz.	9 i więcej godz.
18. Czytał/a Pan/i?<br>☐ TAK Ile razy w ciągu tygodnia?____➡<br>☐ NIE	Ile OGÓŁEM godzin w tygodniu zwykle poświęcał/a Pan/i na to? ➡	Mniej niż 1 godz.	1-2,5 godz.	3-4,5 godz.	5-6,5 godz.	7-8,5 godz.	9 i więcej godz.
19. Wykonywał/a Pan/i ciężkie prace domowe (tj. mycie okien, czyszczenie rynien)?<br>☐ TAK Ile razy w ciągu tygodnia?____➡<br>☐ NIE	Ile OGÓŁEM godzin w tygodniu zwykle poświęcał/a Pan/i na to? ➡	Mniej niż 1 godz.	1-2,5 godz.	3-4,5 godz.	5-6,5 godz.	7-8,5 godz.	9 i więcej godz.
20. Wykonywał/a Pan/i lekkie prace domowe (tj. zamiatanie, odkurzanie)?<br>☐ TAK Ile razy w ciągu tygodnia?____➡<br>☐ NIE	Ile OGÓŁEM godzin w tygodniu zwykle poświęcał/a Pan/i na to? ➡	Mniej niż 1 godz.	1-2,5 godz.	3-4,5 godz.	5-6,5 godz.	7-8,5 godz.	9 i więcej godz.
21.Wykonywał/a Pan/i ciężkie prace ogrodowe (tj. kopanie, grabienie)?<br>☐ TAK Ile razy w ciągu tygodnia?____➡<br>☐ NIE	Ile OGÓŁEM godzin w tygodniu zwykle poświęcał/a Pan/i na to? ➡	Mniej niż 1 godz.	1-2,5 godz.	3-4,5 godz.	5-6,5 godz.	7-8,5 godz.	9 i więcej godz.
22. Wykonywał/a Pan/i lekkie prace ogrodowe (tj. podlewanie roślin)?<br>☐ TAK Ile razy w ciągu tygodnia?____➡<br>☐ NIE	Ile OGÓŁEM godzin w tygodniu zwykle poświęcał/a Pan/i na to? ➡	Mniej niż 1 godz.	1-2,5 godz.	3-4,5 godz.	5-6,5 godz.	7-8,5 godz.	9 i więcej godz.
23. Pracował/a Pan/i przy swoim samochodzie, ciężarówce, kosiarce do trawy lub innym urządzeniu?<br>☐ TAK Ile razy w ciągu tygodnia?____➡<br>☐ NIE	Ile OGÓŁEM godzin w tygodniu zwykle poświęcał/a Pan/i na to? ➡	Mniej niż 1 godz.	1-2,5 godz.	3-4,5 godz.	5-6,5 godz.	7-8,5 godz.	9 i więcej godz.

****Uwaga: Odpowiadając na pytania dotyczące biegania i marszu, proszę uwzględnić ćwiczenia na bieżni mechanicznej.**

24. Biegał/a Pan/i lub uprawiał/a Pan/i jogging?<br>☐ TAK Ile razy w ciągu tygodnia?____➡<br>☐ NIE	Ile OGÓŁEM <u>godzin w tygodniu</u> zwykle poświęcał/a Pan/i na to? ➡	Mniej niż 1 godz.	1-2,5 godz.	3-4,5 godz.	5-6,5 godz.	7-8,5 godz.	9 i więcej godz.
25. Podchodził/a Pan/i pod górę lub wspinał/a się Pan/i (proszę uwzględnić wyłącznie przemieszczanie się pod górę)?<br>☐ TAK Ile razy w ciągu tygodnia?____➡<br>☐ NIE	Ile OGÓŁEM <u>godzin w tygodniu</u> zwykle poświęcał/a Pan/i na to? ➡	Mniej niż 1 godz.	1-2,5 godz.	3-4,5 godz.	5-6,5 godz.	7-8,5 godz.	9 i więcej godz.
26. Spacerował/a Pan/i <u>szybko lub bardzo szybko</u> jako forma ćwiczeń (<u>z wyłączeniem</u> spacerowania w wolnym tempie i wchodzenia pod górę)?<br>☐ TAK Ile razy w ciągu tygodnia?____➡<br>☐ NIE	Ile OGÓŁEM <u>godzin w tygodniu</u> zwykle poświęcał/a Pan/i na to? ➡	Mniej niż 1 godz.	1-2,5 godz.	3-4,5 godz.	5-6,5 godz.	7-8,5 godz.	9 i więcej godz.
27. Chodził/a Pan/i w celu załatwienia różnych spraw (tj. po zakupy, odprowadzanie dzieci do/ze szkoły) (proszę uwzględnić wyłącznie czas poświęcony na <u>chodzenie)</u>?<br>☐ TAK Ile razy w ciągu tygodnia?____➡<br>☐ NIE	Ile OGÓŁEM <u>godzin w tygodniu</u> zwykle poświęcał/a Pan/i na to? ➡	Mniej niż 1 godz.	1-2,5 godz.	3-4,5 godz.	5-6,5 godz.	7-8,5 godz.	9 i więcej godz.
28. Spacerował/a Pan/i <u>w wolnym tempie</u> jako forma ćwiczeń lub dla przyjemności?<br>☐ TAK Ile razy w ciągu tygodnia?____➡<br>☐ NIE	Ile OGÓŁEM <u>godzin w tygodniu</u> zwykle poświęcał/a Pan/i na to? ➡	Mniej niż 1 godz.	1-2,5 godz.	3-4,5 godz.	5-6,5 godz.	7-8,5 godz.	9 i więcej godz.
29. Jeździł/a Pan/i na rowerze lub na rowerze stacjonarnym?<br>☐ TAK Ile razy w ciągu tygodnia?____➡<br>☐ NIE	Ile OGÓŁEM <u>godzin w tygodniu</u> zwykle poświęcał/a Pan/i na to? ➡	Mniej niż 1 godz.	1-2,5 godz.	3-4,5 godz.	5-6,5 godz.	7-8,5 godz.	9 i więcej godz.
30. Ćwiczył/a Pan/i z wykorzystaniem innych urządzeń do treningu tlenowego tj. ergometr, stepper (<u>z wyłączeniem</u> bieżni mechanicznej i roweru stacjonarnego)?<br>☐ TAK Ile razy w ciągu tygodnia?____➡<br>☐ NIE	Ile OGÓŁEM <u>godzin w tygodniu</u> zwykle poświęcał/a Pan/i na to? ➡	Mniej niż 1 godz.	1-2,5 godz.	3-4,5 godz.	5-6,5 godz.	7-8,5 godz.	9 i więcej godz.
31. Wykonywał/a Pan/i ćwiczenia w wodzie (<u>z wyłączeniem</u> pływania)?<br>☐ TAK Ile razy w ciągu tygodnia?____➡<br>☐ NIE	Ile OGÓŁEM <u>godzin w tygodniu</u> zwykle poświęcał/a Pan/i na to? ➡	Mniej niż 1 godz.	1-2,5 godz.	3-4,5 godz.	5-6,5 godz.	7-8,5 godz.	9 i więcej godz.
32. Pływał/a Pan/i w szybkim lub umiarkowanym tempie?<br>☐ TAK Ile razy w ciągu tygodnia?____➡<br>☐ NIE	Ile OGÓŁEM <u>godzin w tygodniu</u> zwykle poświęcał/a Pan/i na to? ➡	Mniej niż 1 godz.	1-2,5 godz.	3-4,5 godz.	5-6,5 godz.	7-8,5 godz.	9 i więcej godz.

33. Pływał/a Pan/i w wolnym tempie?<br>☐ TAK Ile razy w ciągu tygodnia?____ ➡<br>☐ NIE	Ile OGÓŁEM godzin w tygodniu zwykle poświęcał/a Pan/i na to? ➡	Mniej niż 1 godz.	1-2,5 godz.	3-4,5 godz.	5-6,5 godz.	7-8,5 godz.	9 i więcej godz.
34. Wykonywał/a Pan/i ćwiczenia rozciągające lub ćwiczenia gibkości (proszę nie wliczać Jogi lub Tai-chi)?<br>☐ TAK Ile razy w ciągu tygodnia?____ ➡<br>☐ NIE	Ile OGÓŁEM godzin w tygodniu zwykle poświęcał/a Pan/i na to? ➡	Mniej niż 1 godz.	1-2,5 godz.	3-4,5 godz.	5-6,5 godz.	7-8,5 godz.	9 i więcej godz.
35. Ćwiczył/a Pan/i Jogę lub Tai Chi?<br>☐ TAK Ile razy w ciągu tygodnia?____ ➡<br>☐ NIE	Ile OGÓŁEM godzin w tygodniu zwykle poświęcał/a Pan/i na to? ➡	Mniej niż 1 godz.	1-2,5 godz.	3-4,5 godz.	5-6,5 godz.	7-8,5 godz.	9 i więcej godz.
36. Ćwiczył/a Pan/i aerobik lub aerobik taneczny?<br>☐ TAK Ile razy w ciągu tygodnia?____ ➡<br>☐ NIE	Ile OGÓŁEM godzin w tygodniu zwykle poświęcał/a Pan/i na to? ➡	Mniej niż 1 godz.	1-2,5 godz.	3-4,5 godz.	5-6,5 godz.	7-8,5 godz.	9 i więcej godz.
37. Wykonywał/a Pan/i umiarkowany lub intensywny trening siłowy (tj. ćwiczenia z ciężarkami powyżej 2,5 kg, ćwiczenia na siłowni lub pompki)?<br>☐ TAK Ile razy w ciągu tygodnia?____ ➡<br>☐ NIE	Ile OGÓŁEM godzin w tygodniu zwykle poświęcał/a Pan/i na to? ➡	Mniej niż 1 godz.	1-2,5 godz.	3-4,5 godz.	5-6,5 godz.	7-8,5 godz.	9 i więcej godz.
38. Wykonywał/a Pan/i lekki trening siłowy (tj. ćwiczenia z ciężarkami 2,5 kg lub lżejszymi lub ćwiczenia z taśmami elastycznymi)?<br>☐ TAK Ile razy w ciągu tygodnia?____ ➡<br>☐ NIE	Ile OGÓŁEM godzin w tygodniu zwykle poświęcał/a Pan/i na to? ➡	Mniej niż 1 godz.	1-2,5 godz.	3-4,5 godz.	5-6,5 godz.	7-8,5 godz.	9 i więcej godz.
39. Wykonywał/a Pan/i ćwiczenia ogólnorozwojowe, tj. lekkie ćwiczenia gimnastyczne lub ćwiczenia na krześle (z wyłączeniem treningu siłowego)?<br>☐ TAK Ile razy w ciągu tygodnia?____ ➡<br>☐ NIE	Ile OGÓŁEM godzin w tygodniu zwykle poświęcał/a Pan/i na to? ➡	Mniej niż 1 godz.	1-2,5 godz.	3-4,5 godz.	5-6,5 godz.	7-8,5 godz.	9 i więcej godz.
40. Grał/a Pan/i w koszykówkę, piłkę nożną, itp. (z wyłączeniem czasu spędzonego poza boiskiem)?<br>☐ TAK Ile razy w ciągu tygodnia?____ ➡<br>☐ NIE	Ile OGÓŁEM godzin w tygodniu zwykle poświęcał/a Pan/i na to? ➡	Mniej niż 1 godz.	1-2,5 godz.	3-4,5 godz.	5-6,5 godz.	7-8,5 godz.	9 i więcej godz.
41. Podejmował/a Pan/i inne formy aktywności fizycznej nieuwzględnione powyżej (proszę wymienić)<br>________________________<br>☐ TAK Ile razy w ciągu tygodnia?____ ➡<br>☐ NIE	Ile OGÓŁEM godzin w tygodniu zwykle poświęcał/a Pan/i na to? ➡	Mniej niż 1 godz.	1-2,5 godz.	3-4,5 godz.	5-6,5 godz.	7-8,5 godz.	9 i więcej godz.

Dziękujemy za wypełnienie ankiety!
