# Supplementary material for: Validity and Reliability of the Polish Adaptation of the CHAMPS Physical Activity Questionnaire
Source: Biomed Res Int. 2019 Mar 28;2019:6187616. doi: 10.1155/2019/6187616 (PMC6458940; doi:10.1155/2019/6187616)
Supplement: Supplementary Materials — contain Polish version of the CHAMPS physical activity questionnaire. [file 6187616.f1.zip › 6187616.f1/Supplementary materials_BMRI_2704613.doc]

**CHAMPS - KWESTIONARIUSZ AKTYWNOŚCI DLA OSÓB STARSZYCH W POLSKIEJ ADAPTACJI**

Oryginalna wersja:

Data:

Nazwisko lub ID:

CHAMPS: Community Healthy Activities Model Program for Seniors

Institute for Health & Aging, Center for Healthy and Active Aging

University of California, San Francisco

Stanford Center for Research in Disease Prevention, Stanford University

**Ten kwestionariusz dotyczy aktywności, które mógł/a Pan/i podejmować w ciągu ostatnich 4 tygodni. Pytania na kolejnych stronach są podobne do poniższego przykładu.**

**INSTRUKCJA**

**Jeśli PODEJMOWAŁ/A Pan/i określoną aktywność w ciągu ostatnich 4 tygodni:**

**Krok nr 1	Proszę wybrać odpowiedź tak.**

**Krok nr 2	Proszę pomyśleć, ile RAZY w ciągu tygodnia zwykle podejmował/a Pan/i tę aktywność,**

**a następnie wpisać odpowiedź w wyznaczonym miejscu.**

**Krok nr 3	Proszę zaznaczyć, ile GODZIN OGÓŁEM poświęcał/a Pan/i na tę aktywność w ciągu typowego tygodnia.**

**Poniżej przedstawiono przykład odpowiedzi, jaką mogła udzielić Pani Kowalska na Pytanie nr 1:**

Pani Kowalska zwykle odwiedza swoje znajome, Marię i Olgę, dwa razy w tygodniu. Zwykle spędza u Marii 1 godzinę w poniedziałek, a w środę przebywa przez 2 godziny u Olgi. Zatem ogółem pani Kowalska poświęca na spotkania ze znajomymi 3 godziny tygodniowo.

| Czy w ciągu typowego tygodnia podczas ostatnich 4 tygodni… |  | | | | | | |
| --- | --- | --- | --- | --- | --- | --- | --- |
| 1. Odwiedzał/a Pan/i znajomych lub krewnych (z wyłączeniem tych, z którymi Pan/i mieszka)?  TAK Ile razy w ciągu tygodnia? 2  NIE | Ile OGÓŁEM godzin w tygodniu zwykle poświęcał/a Pan/i na to? | Mniej niż 1 godz. | 1-2,5 godz. | 3-4,5 godz. | 5-6,5 godz. | 7-8,5 godz. | 9 i więcej godz. |

**Jeśli NIE PODEJMOWAŁ/A Pan/i danej aktywności:**

- Proszę zaznaczyć odpowiedź NIE i przejść do kolejnego pytania

| Czy w ciągu typowego tygodnia podczas ostatnich 4 tygodni… |  | | | | | | |
| --- | --- | --- | --- | --- | --- | --- | --- |
| 1. Odwiedzał/a Pan/i znajomych lub krewnych (z wyłączeniem tych, z którymi Pan/i mieszka)?  TAK Ile razy w ciągu tygodnia?____  NIE | Ile OGÓŁEM godzin w tygodniu zwykle poświęcał/a Pan/i na to? | Mniej niż 1 godz. | 1-2,5 godz. | 3-4,5 godz. | 5-6,5 godz. | 7-8,5 godz. | 9 i więcej godz. |
| 2. Była Pan/i w ośrodku dla seniorów?  TAK Ile razy w ciągu tygodnia?____  NIE | Ile OGÓŁEM godzin w tygodniu zwykle poświęcał/a Pan/i na to? | Mniej niż 1 godz. | 1-2,5 godz. | 3-4,5 godz. | 5-6,5 godz. | 7-8,5 godz. | 9 i więcej godz. |
| 3. Wykonywał/a Pan/i prace społeczne (wolontariat)?  TAK Ile razy w ciągu tygodnia?____  NIE | Ile OGÓŁEM godzin w tygodniu zwykle poświęcał/a Pan/i na to? | Mniej niż 1 godz. | 1-2,5 godz. | 3-4,5 godz. | 5-6,5 godz. | 7-8,5 godz. | 9 i więcej godz. |
| 4. Był/a Pan/i w kościele lub uczestniczył/a Pan/i w pracach kościelnych?  TAK Ile razy w ciągu tygodnia?____  NIE | Ile OGÓŁEM godzin w tygodniu zwykle poświęcał/a Pan/i na to? | Mniej niż 1 godz. | 1-2,5 godz. | 3-4,5 godz. | 5-6,5 godz. | 7-8,5 godz. | 9 i więcej godz. |
| 5. Był/a Pan/i na spotkaniu jakiegoś klubu lub grupy?  TAK Ile razy w ciągu tygodnia?____  NIE | Ile OGÓŁEM godzin w tygodniu zwykle poświęcał/a Pan/i na to? | Mniej niż 1 godz. | 1-2,5 godz. | 3-4,5 godz. | 5-6,5 godz. | 7-8,5 godz. | 9 i więcej godz. |
| 6. Używał/a Pan/i komputera?  TAK Ile razy w ciągu tygodnia?____  NIE | Ile godzin w tygodniu OGÓŁEM zwykle Pan/i poświęcał/a na to? | Mniej niż 1 godz. | 1-2,5 godz. | 3-4,5 godz. | 5-6,5 godz. | 7-8,5 godz. | 9 i więcej godz. |
| 7. Tańczył/a Pan/i (tj. taniec w parach, w grupie, taniec ludowy, towarzyski) (z wyłączeniem aerobiku tanecznego)?  TAK Ile razy w ciągu tygodnia?____  NIE | Ile OGÓŁEM godzin w tygodniu zwykle poświęcał/a Pan/i na to? | Mniej niż 1 godz. | 1-2,5 godz. | 3-4,5 godz. | 5-6,5 godz. | 7-8,5 godz. | 9 i więcej godz. |
| 8. Zajmował/a się Pan/i stolarką, robótkami ręcznymi, rysowaniem lub inną sztuką czy rzemiosłem?  TAK Ile razy w ciągu tygodnia?____  NIE | Ile OGÓŁEM godzin w tygodniu zwykle poświęcał/a Pan/i na to? | Mniej niż 1 godz. | 1-2,5 godz. | 3-4,5 godz. | 5-6,5 godz. | 7-8,5 godz. | 9 i więcej godz. |
| 9. Grał/a Pan/i w golfa, nosząc lub ciągnąc swój sprzęt golfowy (proszę policzyć wyłącznie czas przeznaczony na chodzenie)?  TAK Ile razy w ciągu tygodnia?____  NIE | Ile OGÓŁEM godzin w tygodniu zwykle poświęcał/a Pan/i na to? | Mniej niż 1 godz. | 1-2,5 godz. | 3-4,5 godz. | 5-6,5 godz. | 7-8,5 godz. | 9 i więcej godz. |
| 10. Grał/a Pan/i w golfa, jeżdżąc wózkiem golfowym (proszę policzyć wyłącznie czas przeznaczony na chodzenie)?  TAK Ile razy w ciągu tygodnia?____  NIE | Ile OGÓŁEM godzin w tygodniu zwykle poświęcał/a Pan/i na to? | Mniej niż 1 godz. | 1-2,5 godz. | 3-4,5 godz. | 5-6,5 godz. | 7-8,5 godz. | 9 i więcej godz. |
| 11. Był/a Pan/i na koncercie, filmie, wykładzie lub wydarzeniu sportowym?  TAK Ile razy w ciągu tygodnia?____  NIE | Ile OGÓŁEM godzin w tygodniu zwykle poświęcał/a Pan/i na to? | Mniej niż 1 godz. | 1-2,5 godz. | 3-4,5 godz. | 5-6,5 godz. | 7-8,5 godz. | 9 i więcej godz. |
| 12. Grał/a Pan/i w karty, bingo lub gry planszowe z innymi osobami?  TAK Ile razy w ciągu tygodnia?____  NIE | Ile OGÓŁEM godzin w tygodniu zwykle poświęcał/a Pan/i na to? | Mniej niż 1 godz. | 1-2,5 godz. | 3-4,5 godz. | 5-6,5 godz. | 7-8,5 godz. | 9 i więcej godz. |
| 13. Grał/a Pan/i w bilard?  TAK Ile razy w ciągu tygodnia?____  NIE | Ile OGÓŁEM godzin w tygodniu zwykle poświęcał/a Pan/i na to? | Mniej niż 1 godz. | 1-2,5 godz. | 3-4,5 godz. | 5-6,5 godz. | 7-8,5 godz. | 9 i więcej godz. |
| 14. Grał/a Pan/i w tenisa w grę pojedynczą (z wyłączeniem gry podwójnej, deblowej)?  TAK Ile razy w ciągu tygodnia?____  NIE | Ile OGÓŁEM godzin w tygodniu zwykle poświęcał/a Pan/i na to? | Mniej niż 1 godz. | 1-2,5 godz. | 3-4,5 godz. | 5-6,5 godz. | 7-8,5 godz. | 9 i więcej godz. |
| 15. Grał/a Pan/i w tenisa w grę podwójną (z wyłączeniem gry pojedynczej, singlowej)?  TAK Ile razy w ciągu tygodnia?____  NIE | Ile OGÓŁEM godzin w tygodniu zwykle poświęcał/a Pan/i na to? | Mniej niż 1 godz. | 1-2,5 godz. | 3-4,5 godz. | 5-6,5 godz. | 7-8,5 godz. | 9 i więcej godz. |
| 16. Jeździł/a Pan/i na łyżwach, wrotkach, rolkach?  TAK Ile razy w ciągu tygodnia?____  NIE | Ile OGÓŁEM godzin w tygodniu zwykle poświęcał/a Pan/i na to? | Mniej niż 1 godz. | 1-2,5 godz. | 3-4,5 godz. | 5-6,5 godz. | 7-8,5 godz. | 9 i więcej godz. |
| 17. Grał/a Pan/i na instrumencie muzycznym?  TAK Ile razy w ciągu tygodnia?____  NIE | Ile OGÓŁEM godzin w tygodniu zwykle poświęcał/a Pan/i na to? | Mniej niż 1 godz. | 1-2,5 godz. | 3-4,5 godz. | 5-6,5 godz. | 7-8,5 godz. | 9 i więcej godz. |
| 18. Czytał/a Pan/i?  TAK Ile razy w ciągu tygodnia?____  NIE | Ile OGÓŁEM godzin w tygodniu zwykle poświęcał/a Pan/i na to? | Mniej niż 1 godz. | 1-2,5 godz. | 3-4,5 godz. | 5-6,5 godz. | 7-8,5 godz. | 9 i więcej godz. |
| 19. Wykonywał/a Pan/i ciężkie prace domowe (tj. mycie okien, czyszczenie rynien)?  TAK Ile razy w ciągu tygodnia?____  NIE | Ile OGÓŁEM godzin w tygodniu zwykle poświęcał/a Pan/i na to? | Mniej niż 1 godz. | 1-2,5 godz. | 3-4,5 godz. | 5-6,5 godz. | 7-8,5 godz. | 9 i więcej godz. |
| 20. Wykonywał/a Pan/i lekkie prace domowe (tj. zamiatanie, odkurzanie)?  TAK Ile razy w ciągu tygodnia?____  NIE | Ile OGÓŁEM godzin w tygodniu zwykle poświęcał/a Pan/i na to? | Mniej niż 1 godz. | 1-2,5 godz. | 3-4,5 godz. | 5-6,5 godz. | 7-8,5 godz. | 9 i więcej godz. |
| 21.Wykonywał/a Pan/i ciężkie prace ogrodowe (tj. kopanie, grabienie)?  TAK Ile razy w ciągu tygodnia?____  NIE | Ile OGÓŁEM godzin w tygodniu zwykle poświęcał/a Pan/i na to? | Mniej niż 1 godz. | 1-2,5 godz. | 3-4,5 godz. | 5-6,5 godz. | 7-8,5 godz. | 9 i więcej godz. |
| 22. Wykonywał/a Pan/i lekkie prace ogrodowe (tj. podlewanie roślin)?  TAK Ile razy w ciągu tygodnia?____  NIE | Ile OGÓŁEM godzin w tygodniu zwykle poświęcał/a Pan/i na to? | Mniej niż 1 godz. | 1-2,5 godz. | 3-4,5 godz. | 5-6,5 godz. | 7-8,5 godz. | 9 i więcej godz. |
| 23. Pracował/a Pan/i przy swoim samochodzie, ciężarówce, kosiarce do trawy lub innym urządzeniu?  TAK Ile razy w ciągu tygodnia?____  NIE | Ile OGÓŁEM godzin w tygodniu zwykle poświęcał/a Pan/i na to? | Mniej niż 1 godz. | 1-2,5 godz. | 3-4,5 godz. | 5-6,5 godz. | 7-8,5 godz. | 9 i więcej godz. |
| ****Uwaga: Odpowiadając na pytania dotyczące biegania i marszu, proszę uwzględnić ćwiczenia na bieżni mechanicznej.** | | | | | | | |
| 24. Biegał/a Pan/i lub uprawiał/a Pan/i jogging?  TAK Ile razy w ciągu tygodnia?____  NIE | Ile OGÓŁEM godzin w tygodniu zwykle poświęcał/a Pan/i na to? | Mniej niż 1 godz. | 1-2,5 godz. | 3-4,5 godz. | 5-6,5 godz. | 7-8,5 godz. | 9 i więcej godz. |
| 25. Podchodził/a Pan/i pod górę lub wspinał/a się Pan/i (proszę uwzględnić wyłącznie przemieszczanie się pod górę)?  TAK Ile razy w ciągu tygodnia?____  NIE | Ile OGÓŁEM godzin w tygodniu zwykle poświęcał/a Pan/i na to? | Mniej niż 1 godz. | 1-2,5 godz. | 3-4,5 godz. | 5-6,5 godz. | 7-8,5 godz. | 9 i więcej godz. |
| 26. Spacerował/a Pan/i szybko lub bardzo szybko jako forma ćwiczeń (z wyłączeniem spacerowania w wolnym tempie i wchodzenia pod górę)?  TAK Ile razy w ciągu tygodnia?____  NIE | Ile OGÓŁEM godzin w tygodniu zwykle poświęcał/a Pan/i na to? | Mniej niż 1 godz. | 1-2,5 godz. | 3-4,5 godz. | 5-6,5 godz. | 7-8,5 godz. | 9 i więcej godz. |
| 27. Chodził/a Pan/i w celu załatwienia różnych spraw (tj. po zakupy, odprowadzanie dzieci do/ze szkoły) (proszę uwzględnić wyłącznie czas poświęcony na chodzenie)?  TAK Ile razy w ciągu tygodnia?____  NIE | Ile OGÓŁEM godzin w tygodniu zwykle poświęcał/a Pan/i na to? | Mniej niż 1 godz. | 1-2,5 godz. | 3-4,5 godz. | 5-6,5 godz. | 7-8,5 godz. | 9 i więcej godz. |
| 28. Spacerował/a Pan/i w wolnym tempie jako forma ćwiczeń lub dla przyjemności?  TAK Ile razy w ciągu tygodnia?____  NIE | Ile OGÓŁEM godzin w tygodniu zwykle poświęcał/a Pan/i na to? | Mniej niż 1 godz. | 1-2,5 godz. | 3-4,5 godz. | 5-6,5 godz. | 7-8,5 godz. | 9 i więcej godz. |
| 29. Jeździł/a Pan/i na rowerze lub na rowerze stacjonarnym?  TAK Ile razy w ciągu tygodnia?____  NIE | Ile OGÓŁEM godzin w tygodniu zwykle poświęcał/a Pan/i na to? | Mniej niż 1 godz. | 1-2,5 godz. | 3-4,5 godz. | 5-6,5 godz. | 7-8,5 godz. | 9 i więcej godz. |
| 30. Ćwiczył/a Pan/i z wykorzystaniem innych urządzeń do treningu tlenowego tj. ergometr, stepper (z wyłączeniem bieżni mechanicznej i roweru stacjonarnego)?  TAK Ile razy w ciągu tygodnia?____  NIE | Ile OGÓŁEM godzin w tygodniu zwykle poświęcał/a Pan/i na to? | Mniej niż 1 godz. | 1-2,5 godz. | 3-4,5 godz. | 5-6,5 godz. | 7-8,5 godz. | 9 i więcej godz. |
| 31. Wykonywał/a Pan/i ćwiczenia w wodzie (z wyłączeniem pływania)?  TAK Ile razy w ciągu tygodnia?____  NIE | Ile OGÓŁEM godzin w tygodniu zwykle poświęcał/a Pan/i na to? | Mniej niż 1 godz. | 1-2,5 godz. | 3-4,5 godz. | 5-6,5 godz. | 7-8,5 godz. | 9 i więcej godz. |
| 32. Pływał/a Pan/i w szybkim lub umiarkowanym tempie?  TAK Ile razy w ciągu tygodnia?____  NIE | Ile OGÓŁEM godzin w tygodniu zwykle poświęcał/a Pan/i na to? | Mniej niż 1 godz. | 1-2,5 godz. | 3-4,5 godz. | 5-6,5 godz. | 7-8,5 godz. | 9 i więcej godz. |
| 33. Pływał/a Pan/i w wolnym tempie?  TAK Ile razy w ciągu tygodnia?____  NIE | Ile OGÓŁEM godzin w tygodniu zwykle poświęcał/a Pan/i na to? | Mniej niż 1 godz. | 1-2,5 godz. | 3-4,5 godz. | 5-6,5 godz. | 7-8,5 godz. | 9 i więcej godz. |
| 34. Wykonywał/a Pan/i ćwiczenia rozciągające lub ćwiczenia gibkości (proszę nie wliczać Jogi lub Tai-chi)?  TAK Ile razy w ciągu tygodnia?____  NIE | Ile OGÓŁEM godzin w tygodniu zwykle poświęcał/a Pan/i na to? | Mniej niż 1 godz. | 1-2,5 godz. | 3-4,5 godz. | 5-6,5 godz. | 7-8,5 godz. | 9 i więcej godz. |
| 35. Ćwiczył/a Pan/i Jogę lub Tai Chi?  TAK Ile razy w ciągu tygodnia?____  NIE | Ile OGÓŁEM godzin w tygodniu zwykle poświęcał/a Pan/i na to? | Mniej niż 1 godz. | 1-2,5 godz. | 3-4,5 godz. | 5-6,5 godz. | 7-8,5 godz. | 9 i więcej godz. |
| 36. Ćwiczył/a Pan/i aerobik lub aerobik taneczny?  TAK Ile razy w ciągu tygodnia?____  NIE | Ile OGÓŁEM godzin w tygodniu zwykle poświęcał/a Pan/i na to? | Mniej niż 1 godz. | 1-2,5 godz. | 3-4,5 godz. | 5-6,5 godz. | 7-8,5 godz. | 9 i więcej godz. |
| 37. Wykonywał/a Pan/i umiarkowany lub intensywny trening siłowy (tj. ćwiczenia z ciężarkami powyżej 2,5 kg, ćwiczenia na siłowni lub pompki)?  TAK Ile razy w ciągu tygodnia?____  NIE | Ile OGÓŁEM godzin w tygodniu zwykle poświęcał/a Pan/i na to? | Mniej niż 1 godz. | 1-2,5 godz. | 3-4,5 godz. | 5-6,5 godz. | 7-8,5 godz. | 9 i więcej godz. |
| 38. Wykonywał/a Pan/i lekki trening siłowy (tj. ćwiczenia z ciężarkami 2,5 kg lub lżejszymi lub ćwiczenia z taśmami elastycznymi)?  TAK Ile razy w ciągu tygodnia?____  NIE | Ile OGÓŁEM godzin w tygodniu zwykle poświęcał/a Pan/i na to? | Mniej niż 1 godz. | 1-2,5 godz. | 3-4,5 godz. | 5-6,5 godz. | 7-8,5 godz. | 9 i więcej godz. |
| 39. Wykonywał/a Pan/i ćwiczenia ogólnorozwojowe, tj. lekkie ćwiczenia gimnastyczne lub ćwiczenia na krześle (z wyłączeniem treningu siłowego)?  TAK Ile razy w ciągu tygodnia?____  NIE | Ile OGÓŁEM godzin w tygodniu zwykle poświęcał/a Pan/i na to? | Mniej niż 1 godz. | 1-2,5 godz. | 3-4,5 godz. | 5-6,5 godz. | 7-8,5 godz. | 9 i więcej godz. |
| 40. Grał/a Pan/i w koszykówkę, piłkę nożną, itp. (z wyłączeniem czasu spędzonego poza boiskiem)?  TAK Ile razy w ciągu tygodnia?____  NIE | Ile OGÓŁEM godzin w tygodniu zwykle poświęcał/a Pan/i na to? | Mniej niż 1 godz. | 1-2,5 godz. | 3-4,5 godz. | 5-6,5 godz. | 7-8,5 godz. | 9 i więcej godz. |
| 41. Podejmował/a Pan/i inne formy aktywności fizycznej nieuwzględnione powyżej (proszę wymienić)  TAK Ile razy w ciągu tygodnia?____  NIE | Ile OGÓŁEM godzin w tygodniu zwykle poświęcał/a Pan/i na to? | Mniej niż 1 godz. | 1-2,5 godz. | 3-4,5 godz. | 5-6,5 godz. | 7-8,5 godz. | 9 i więcej godz. |

***Dziękujemy za wypełnienie ankiety!***
